# Supplementary material for: When the going gets tough, the entrepreneurs get less entrepreneurial?
Source: PLoS One. 2023 Dec 22;18(12):e0290253. doi: 10.1371/journal.pone.0290253 (PMC10745155; doi:10.1371/journal.pone.0290253)
Supplement: S1 File — (DOCX) [file pone.0290253.s001.docx]

# Supporting information

## Sample selection analysis

The idea behind this analysis is that recruited ventures fall in between the full population and volunteering ventures. Thus, where volunteers differ from recruited ventures, our sample would differ from the full population on that variable and in that same direction. Likewise, this intuition is used to assess sample selection in surveys; check differences between early and late responders based on the assumption that late responders are in between the full population and the early responders (Lin & Schaeffer, 1995).

#### S1 Table. Hard times in terms of profit by recruited vs. volunteered. *n*=177

|  | Crisis times |  | Recovery times |  |
| --- | --- | --- | --- | --- |
|  | Recruited | Volunteered | Recruited | Volunteered |
| Neutral | 57 | 58 | 56 | 51 |
| Somewhat hard | 15 | 15 | 17 | 16 |
| Hard | 9 | 6 | 12 | 9 |
| Very hard | 11 | 6 | 7 | 9 |

#### S2 Table. Hard times in terms of employees by recruited vs. volunteered. *n*=177

|  | Crisis times |  | Recovery times |  |
| --- | --- | --- | --- | --- |
|  | Recruited | Volunteered | Recruited | Volunteered |
| Neutral | 77 | 72 | 77 | 68 |
| Somewhat hard | 8 | 4 | 9 | 7 |
| Hard | 4 | 6 | 5 | 8 |
| Very hard | 3 | 3 | 1 | 2 |

#### S3 Table. Recruited vs. volunteered correlation with SJS, ESE, and EO. *n*=177

|  | SJS | ESE | EO |
| --- | --- | --- | --- |
| I experience in my job…job security | -.02 |  |  |
| Deal effectively with day-to-day problems and crises |  | -.08 |  |
| Delegate tasks and responsibilities to employees in my business |  | .11 |  |
| Proactiveness |  |  | -.19 |
| Innovativeness |  |  | -.18 |
| Risk taking |  |  | -.15 |

SJS and ESE are largely uncorrelated with volunteering. Individual ESE items show mostly small positive and negative correlations that balance each other out, even within subdimensions. Table A3 shows the most extreme correlations.

All three EO subdimensions show strong negative correlations with volunteering. This means that high EO ventures are less likely to volunteer for the consulting program. Thus, the range of the dependent variable in our sample may be restricted compared to the full population. This could bias results if the strength of effects would differ along the range of EO. However, we observe no such nonlinearities in our visualizations of the data (e.g., Figs 9-11). So, the main consequence of this selection is one of external validity. Specifically, effect sizes scale with the standard deviation of the dependent variable, such that if the range is restricted, estimated effect sizes should be smaller and thus our study may only be more conservative, if anything.

## Measurement details

### Hard times

We measure hard times in four ways: (1) during the crisis and regarding a) profit and b) number of employees, and (2) after the crisis regarding a) profit and b) number of employees. We start with the following satisfaction measures:

To what extent are you satisfied with the average business outcomes during the crisis years?

Profit (1-7)

Number of employees (1-7)

To what extent are you satisfied with the average business outcomes in the period after the crisis?

Profit (1-7)

Number of employees (1-7)

The responses are then clustered and reversed:

1 (Neutral or satisfied) 2 (somewhat dissatisfied) 3 (dissatisfied) 4 (very dissatisfied).

We relabel those options to:

1 (Neutral or better) 2 (Somewhat hard) 3 (Hard) 4 (Very hard)

The crisis does not imply hard times for all firms. Some firms may be resilient during the crisis or even use the crisis as an opportunity. Our measure sensibly classifies their entrepreneurs as experiencing neutral or better times.

Some dissatisfied entrepreneurs may have been dissatisfied already before the crisis. Our measure classifies them as experiencing hard times. That is sensible because responses are likely context dependent. Low performance during a crisis which is as low as the performance last year without a crisis may be viewed as neutral in hindsight, so a dissatisfied response should mean especially low performance, so it deserves to be classified as experiencing hard times.

We include dissatisfaction with the number of employees not only to do justice to ventures being about more than profit, but specifically also because we expect dissatisfaction with the number of employees to reflect entrepreneurs being forced to fire people due to the crisis. In small ventures, firing an employee can be a tough experience for the entrepreneur that we expect to affect their cognitions and affects about their venture. Losing employees to the crisis should be a concrete and powerful instance of the venture’s fragility. By contrast, losing profit is “only on paper” and does not reflect the core of the venture like losing one of “your people”.

### Sense of job security

Sense of job security was assessed in as part of a list of experiences on the job:

I experience in my job…

job security (1-3)

The SJS measure is positively phrased as opposed to measures of (sense of) job insecurity. The literature has not yet settled on a preference regarding such coding (Shoss, 2017). Especially for entrepreneurs, it may be easier to indicate low SJS than high sense of job insecurity, such that our phrasing increases response variety. Second, the measure is a single item, as opposed to exploring SJS as multidimensional. Multidimensional measures can be significantly related to antecedents while single items measures are not (e.g., age), but relationships to antecedents are not significantly different between measurement approaches (Keim et al., 2014). Third, the measure asks about respondent’s SJS in an overall and direct way, rather than asking for one or more specific aspects of a theoretical definition of job security. Shoss (2017, p. 1920) identifies overall perception measures as preferable to composite scales. Still, one drawback of such a measure is that the measure depends on respondents’ interpretation of the concept. For example, the measure may reflect affective components for some respondents, while reflecting only the cognitive component (i.e., estimates of likelihood of losing the job in the next year or two) for other respondents.

### Expectation/value of job security

The extent to which respondents value job security was assessed as part of a list of values regarding work:

From my work/job I expect/value…

job security (1-5)

We mention both expectation and value here to better capture the meaning of the Dutch word used in the survey.

## Correlations

#### S4 Table. Correlations

|  | 1 | 2 | 3 | 4 | 5 | 6 | 7 | 8 | 9 | 10 | 11 | 12 | 13 |  |
| --- | --- | --- | --- | --- | --- | --- | --- | --- | --- | --- | --- | --- | --- | --- |
|  | | | | | | | | | | | | | | |
| 1. Hard crisis times (# of employees) |  |  |  |  |  |  |  |  |  |  |  |  |  |  |
| 2. Hard crisis times (profit) | .429 |  |  |  |  |  |  |  |  |  |  |  |  |  |
| 3. Hard recovery times (# of employees) | .531 | .180 |  |  |  |  |  |  |  |  |  |  |  |  |
| 4. Hard recovery times (profit) | .225 | .520 | .325 |  |  |  |  |  |  |  |  |  |  |  |
| 5. Profit | -.052 | -.178 | -.088 | -.315 |  |  |  |  |  |  |  |  |  |  |
| 6. Profit growth | -.002 | -.041 | -.055 | -.225 | .236 |  |  |  |  |  |  |  |  |  |
| 7. Profit instability | .037 | -.010 | -.045 | .033 | -.013 | -.006 |  |  |  |  |  |  |  |  |
| 8. Firm size | .139 | .158 | .074 | .084 | .052 | -.058 | .216 |  |  |  |  |  |  |  |
| 9. Tenure | .085 | .161 | .016 | .109 | -.060 | -.092 | .010 | .127 |  |  |  |  |  |  |
| 10. Need for achievement | .032 | .065 | .030 | .022 | .006 | -.009 | -.092 | -.085 | -.067 |  |  |  |  |  |
| 11. Job security value | -.058 | -.051 | -.038 | -.045 | -.050 | -.182 | .012 | .004 | .074 | -.073 |  |  |  |  |
| 12. Entrepreneurial self-efficacy | -.000 | -.013 | -.079 | -.104 | .042 | .040 | -.020 | .069 | -.102 | .452 | -.036 |  |  |  |
| 13. Sense of job security | -.136 | -.205 | -.154 | -.285 | .019 | .079 | -.057 | .012 | .051 | .040 | .208 | .174 |  |  |
| 14. Entrepreneurial orientation | -.077 | -.056 | -.164 | -.076 | .089 | .109 | .002 | .071 | -.082 | .302 | .032 | .280 | .129 |  |

## Entrepreneurial self-efficacy factor analysis

#### S5 Table. Entrepreneurial self-efficacy loadings

| Item  (How much confidence do you have in your ability to…) | Factor 1 | Factor 2 | Factor 3 | Factor 4 | Factor 5 |
| --- | --- | --- | --- | --- | --- |
| Brainstorm a new idea for a product/service |  |  | .759 |  |  |
| Identify the need for a new product/service |  |  | .754 |  |  |
| Design a product/service that will satisfy customer needs and wants |  |  | .802 |  |  |
| Determine a competitive price for a new product or service |  |  | .134 | .104 | .564 |
| Estimate the amount of start-up funds and working capital necessary to start my business |  |  |  |  | .795 |
| Get others to identify with and believe in my vision and plans for a new business |  |  | .112 | .656 |  |
| Network—i.e., make contact with and exchange information with others |  |  |  | .594 | -.140 |
| Clearly and concisely explain verbally/in writing my business idea in everyday terms |  |  | -.127 | .650 | .215 |
| Supervise employees |  | .715 |  |  |  |
| Recruit and hire employees | -.110 | .662 | -.101 |  | .135 |
| Delegate tasks and responsibilities to employees in my business | .109 | .682 | .108 | -.113 |  |
| Deal effectively with day-to-day problems and crises |  | .511 | .107 |  | -.102 |
| Train employees |  | .534 |  | .170 |  |
| Organize and maintain the financial records of my business | .872 |  |  |  |  |
| Manage the financial assets of my busines | .910 |  |  |  |  |
| Read and interpret financial statements | .667 |  |  |  | .178 |

We use factor analysis with promax rotation that predefines five factors (see also McGee & Peterson, 2019). All items load with the other items of their subdimension. We prune planning1, planning4, and implementing-people5 due to .51, 37, and .33 cross-loadings with searching, marshalling, and marshalling, respectively. Then, the model fits the data well with clean loadings and 52% of variance explained (although the model explained 50% of variance before pruning, and a few items still have uniqueness greater than 0.5).

## Entrepreneurial orientation factor analysis

#### S6 Table. Entrepreneurial orientation loadings

| Item | Factor1 | Factor 2 | Factor 3 |
| --- | --- | --- | --- |
| In general, the venture emphasizes…  existing products or services vs.  R&D, technological leadership and innovation | .101 | .299 |  |
| In the last three years, the venture has brought to the market…  no new products or services vs. many new products or services |  | .887 |  |
| In the last three years, changes to products or services were… limited vs. radical | .100 | .735 |  |
| When dealing with (potential competitors), the venture…  Typically responds to actions which competitors initiate vs.  Typically initiates actions to which competitors then respond |  |  | .546 |
| In dealing with (potential) competitors, the venture typically…  is rarely the first venture to introduce new products, services, administrative techniques, operational technologies etc. vs.  is very often the first venture to introduce [such things]. |  |  | .850 |
| In dealing with (potential) competitors, the venture…  Typically seeks to avoid competitive clashes, preferring a “live-and-let-live” posture vs.  Typically adopts a very competitive, “undo-the-competitors” posture | .208 |  | .577 |
| In general the venture tends toward…  low-risk projects (with normal and certain rates of return) vs.  high-risk projects (with chances of very high returns) | .697 |  |  |
| Owing to the nature of the environment, the venture believes… it is best to explore it gradually via cautious, incremental behavior vs.  bold, wide-ranging acts are necessary to achieve the firm’s objectives | .859 |  |  |
| When confronted with decision-making situations involving uncertainty, the venture . . .  Typically adopts a cautious, “wait-and-see” posture in order to minimize the probability of making costly decisions vs.  Typically adopts a bold, aggressive posture in order to maximize the probability of exploiting potential opportunities | .732 |  |  |

The factor analysis with promax rotation and a predefined three factors shows a good fit, except for innovativeness1. It loads weakly only because the vast majority responded 1 out of 5. However, we believe it is valuable to include the information from items that discriminate a more extreme range such as innovativeness1. Correlational analysis such as factor analyses undervalue such information. The final score is calculated by multiplying the loading-weighted averages of the subdimensions.

## Full structural equation model (SEM) estimation output

#### S7 Table. SEM output for hard crisis times in terms of number of employees. *n*=247

|  | Coefficient | Standard error  (Sandwich) | Standardized effect size |
| --- | --- | --- | --- |
| *Sense of job security* |  |  |  |
| Hard times | -.15 | .07 | .13 |
| Profit | -.000 | .005 | -.003 |
| Profit growth | .021 | .011 | .133 |
| Profit instability | -.011 | .013 | -.058 |
| Firm size | .031 | .041 | .048 |
| Tenure | .004 | .005 | .054 |
| Job security value | .15 | .04 | .23 |
| Need for achievement | .069 | .062 | .067 |
|  |  |  |  |
| *Entrepreneurial self-efficacy* |  |  |  |
| Hard times | -3.26 | 6.29 | -.02 |
| Profit | .143 | .494 | .014 |
| Profit growth | .805 | 1.08 | .041 |
| Profit instability | -.081 | 1.39 | -.003 |
| Firm size | 9.15 | 4.71 | .118 |
| Tenure | -.785 | .497 | -.086 |
| Job security value | 1.22 | 5.69 | .016 |
| Need for achievement | 56.32 | 7.90 | .46 |
|  |  |  |  |
| *Entrepreneurial orientation* |  |  |  |
| Sense of job security | 1.54 | 1.60 | .06 |
| Entrepreneurial self-efficacy | .03 | .017 | .13 |
| Hard crisis times (profit) | -2.22 | 1.37 | -.08 |
| Profit | .100 | .119 | .049 |
| Profit growth | .404 | .235 | .101 |
| Profit instability | .066 | .300 | .014 |
| Firm size | 1.68 | 1.10 | .105 |
| Tenure | -.091 | .105 | -.048 |
| Job security value | .899 | 1.14 | .056 |
| Need for achievement | 6.57 | 1.68 | .26 |
|  |  |  |  |
| *Indirect effects* |  |  |  |
| Via sense of job security | -.23 | .27 | -.008 |
| Via entrepreneurial self-efficacy | -.09 | .17 | -.003 |
| *Total hard times effect* | -2.54 | 1.43 | -.09 |

#### S8 Table. SEM output for hard crisis times in terms of profit. *n*=248

|  | Coefficient | Standard error  (Sandwich) | Standardized effect size |
| --- | --- | --- | --- |
| *Sense of job security* |  |  |  |
| Hard times | -.16 | .05 | -.23 |
| Profit | -.004 | .005 | -.045 |
| Profit growth | .021 | .011 | .131 |
| Profit instability | -.014 | .013 | -.073 |
| Firm size | .042 | .042 | .066 |
| Tenure | .006 | .005 | .073 |
| Job security value | .14 | .04 | .23 |
| Need for achievement | .082 | .062 | .080 |
|  |  |  |  |
| *Entrepreneurial self-efficacy* |  |  |  |
| Hard times | -4.68 | 4.16 | -.05 |
| Profit | -.209 | .564 | -.021 |
| Profit growth | .660 | 1.094 | .033 |
| Profit instability | -.619 | 1.458 | -.026 |
| Firm size | 9.37 | 4.70 | .12 |
| Tenure | -.862 | .505 | -.092 |
| Job security value | 2.175 | 5.758 | .027 |
| Need for achievement | 58.80 | 8.12 | .46 |
|  |  |  |  |
| *Entrepreneurial orientation* |  |  |  |
| Sense of job security | 1.54 | 1.58 | .06 |
| Entrepreneurial self-efficacy | .025 | .016 | .12 |
| Hard times | -.86 | .995 | -.05 |
| Profit | .103 | .118 | .051 |
| Profit growth | .410 | .233 | .102 |
| Profit instability | .075 | .297 | .016 |
| Firm size | 1.66 | 1.08 | .104 |
| Tenure | -.085 | 0.11 | -.045 |
| Job security value | .874 | 1.13 | .055 |
| Need for achievement | 6.61 | 1.69 | .26 |
|  |  |  |  |
| *Indirect effects* |  |  |  |
| Via sense of job security | -.25 | .26 | .014 |
| Via entrepreneurial self-efficacy | -.12 | .12 | .007 |
| *Total hard times effect* | -1.22 | 1.04 | -.07 |

#### S9 Table. SEM output for hard recovery times in terms of number of employees. *n*=248

|  | Coefficient | Standard error  (Sandwich) | Standardized effect size |
| --- | --- | --- | --- |
| *Sense of job security* |  |  |  |
| Hard times | -.17 | .07 | -.15 |
| Profit | -.001 | .005 | -.017 |
| Profit growth | .021 | .010 | .127 |
| Profit instability | -.014 | .013 | -.069 |
| Firm size | .024 | .041 | .037 |
| Tenure | .003 | .005 | .038 |
| Job security value | .15 | .04 | .23 |
| Need for achievement | .059 | .063 | .057 |
|  |  |  |  |
| *Entrepreneurial self-efficacy* |  |  |  |
| Hard times | -14.38 | 6.40 | -.10 |
| Profit | -.145 | .572 | -.014 |
| Profit growth | .629 | 1.09 | .031 |
| Profit instability | -.675 | 1.45 | -.028 |
| Firm size | 9.56 | 4.67 | .120 |
| Tenure | -.929 | .507 | -.099 |
| Job security value | 2.10 | 5.69 | .026 |
| Need for achievement | 59.10 | 8.02 | .47 |
|  |  |  |  |
| *Entrepreneurial orientation* |  |  |  |
| Sense of job security | 1.19 | 1.56 | .05 |
| Entrepreneurial self-efficacy | .022 | .016 | .11 |
| Hard times | -4.14 | 1.29 | -.15 |
| Profit | .092 | .121 | .045 |
| Profit growth | .377 | .236 | .094 |
| Profit instability | .016 | .289 | .003 |
| Firm size | 1.83 | 1.09 | .115 |
| Tenure | -.094 | .103 | -.050 |
| Job security value | .849 | 1.10 | .053 |
| Need for achievement | 6.91 | 1.68 | .27 |
|  |  |  |  |
| *Indirect effects* |  |  |  |
| Via sense of job security | -.20 | .29 | -.007 |
| Via entrepreneurial self-efficacy | -.32 | .25 | -.011 |
| *Total hard times effect* | -4.67 | 1.30 | -.17 |

#### S10 Table. SEM output for hard recovery times in terms of profit. *n*=248

|  | Coefficient | Standard error  (Sandwich) | Standardized effect size |
| --- | --- | --- | --- |
| *Sense of job security* |  |  |  |
| Hard times | -.19 | .05 | -.29 |
| Profit | -.007 | .005 | -.083 |
| Profit growth | .014 | .011 | .089 |
| Profit instability | -.010 | .012 | -.052 |
| Firm size | .030 | .039 | .047 |
| Tenure | .005 | .004 | .062 |
| Job security value | .135 | .040 | .209 |
| Need for achievement | .058 | .061 | .057 |
|  |  |  |  |
| *Entrepreneurial self-efficacy* |  |  |  |
| Hard times | -10.43 | 4.49 | -.13 |
| Profit | -.489 | .584 | -.048 |
| Profit growth | .240 | 1.07 | .012 |
| Profit instability | -.471 | 1.46 | -.019 |
| Firm size | 9.60 | 4.59 | .120 |
| Tenure | -.812 | .496 | -.086 |
| Job security value | 1.08 | 5.76 | .014 |
| Need for achievement | 58.30 | 7.92 | .46 |
|  |  |  |  |
| *Entrepreneurial orientation* |  |  |  |
| Sense of job security | 1.70 | 1.68 | .07 |
| Entrepreneurial self-efficacy | .026 | .016 | .13 |
| Hard times | -.20 | .92 | -.012 |
| Profit | .122 | .127 | .060 |
| Profit growth | .389 | .235 | .097 |
| Profit instability | .074 | .292 | .015 |
| Firm size | 1.537 | 1.074 | .096 |
| Tenure | -.099 | .103 | -.053 |
| Job security value | .956 | 1.13 | .060 |
| Need for achievement | 6.60 | 1.67 | .26 |
|  |  |  |  |
| *Indirect effects* |  |  |  |
| Via sense of job security | -.32 | .34 | -.020 |
| Via entrepreneurial self-efficacy | -.27 | .19 | -.016 |
| *Total hard times effect* | -.80 | .87 | -.05 |

## Primer on partial regression plots

The x-axis of the partial regression plots shows the residuals from a linear regression of ESE (or SJS) on all covariates except hard times. The y-axis gives the residuals from a linear regression of EO on all covariates except hard times, ESE and SJS. Therefore, the plot visualizes the relationship between X and Y after the covariates are partialled out, like a multivariate regression model partials out covariates in the coefficient estimate (see Moya-Laraño & Corcobado, 2008, for more methodological details).

To visualize the mediation, the covariation with hard times must be part of the residuals of ESE and SJS. Therefore, we also need to exclude hard times from the regression on those mediators and EO. Then, each observation gets a different shape and color depending on their value on hard times. The data clouds are the same for all four types of hard times in terms of location. The only difference between plots is that some observations differ in shape-color if they have different values on different types of hard times.

## Brief R script

library(lavaan)

modelcp <- '

Jobsecurity ~ a*hardcrisistimesprofit + ihsprofit + ihspgrowth + lpu + PersoneelsklasseBTW_O1 + tenure + wwjobsecurity + Need.for.Achievement

Entrepreneurial.self.efficacy ~ b*hardcrisistimesprofit + ihsprofit + ihspgrowth + lpu + PersoneelsklasseBTW_O1 + tenure+ wwjobsecurity + Need.for.Achievement

Entrepreneurial.orientation ~ c*Jobsecurity + d*Entrepreneurial.self.efficacy + e*hardcrisistimesprofit + ihsprofit + ihspgrowth + lpu + PersoneelsklasseBTW_O1 + tenure+ wwjobsecurity + Need.for.Achievement

Indirect effect via SJS := a*c

Indirect effect via ESE := b*d

Total effect := a*c + b*d + e

'

## ihsprofit= inverse hyperbolic sine transformation of profit

## ihspgrowth = inverse hyperbolic sine transformation of profit growth

## lpu = log of profit instability

## PersoneelsklasseBTW_O1 = firm size

## wwjobsecurity = job security value

fitcp<- sem(modelcp, data = maindf)

summary(fitcp, standardized=TRUE)

modelrp <- '

Jobsecurity ~ a*hardrecoverytimesprofit + ihsprofit + ihspgrowth + lpu + PersoneelsklasseBTW_O1 + tenure + wwjobsecurity + Need.for.Achievement

Entrepreneurial.self.efficacy ~ b*hardrecoverytimesprofit + ihsprofit + ihspgrowth + lpu + PersoneelsklasseBTW_O1 + tenure + wwjobsecurity + Need.for.Achievement

Entrepreneurial.orientation ~ c*Jobsecurity + d*Entrepreneurial.self.efficacy + e*hardrecoverytimesprofit + ihsprofit + ihspgrowth + lpu + PersoneelsklasseBTW_O1 + tenure+ wwjobsecurity + Need.for.Achievement

Indirect effect via SJS := a*c

Indirect effect via ESE := b*d

Total effect := a*c + b*d + e

'

fitrp<- sem(modelrp, data = maindf)

summary(fitrp, standardized=TRUE)

modelce <- '

Jobsecurity ~ a*hardcrisistimesemployees + ihsprofit + ihspgrowth + lpu + PersoneelsklasseBTW_O1 + tenure + wwjobsecurity + Need.for.Achievement

Entrepreneurial.self.efficacy ~ b*hardcrisistimesemployees + ihsprofit + ihspgrowth + lpu + PersoneelsklasseBTW_O1 + tenure+ wwjobsecurity + Need.for.Achievement

Entrepreneurial.orientation ~ c*Jobsecurity + d*Entrepreneurial.self.efficacy + e*hardcrisistimesemployees + ihsprofit + ihspgrowth + lpu + PersoneelsklasseBTW_O1 + tenure + wwjobsecurity + Need.for.Achievement

Indirect effect via SJS := a*c

Indirect effect via ESE := b*d

Total effect := a*c + b*d + e

'

fitce<- sem(modelce, data = maindf)

summary(fitce, standardized=TRUE)

modelre <- '

Jobsecurity ~ a*hardrecoverytimesemployees + ihsprofit + ihspgrowth + lpu + PersoneelsklasseBTW_O1 + tenure + wwjobsecurity + Need.for.Achievement

Entrepreneurial.self.efficacy ~ b*hardrecoverytimesemployees + ihsprofit + ihspgrowth + lpu + PersoneelsklasseBTW_O1 + tenure + wwjobsecurity + Need.for.Achievement

Entrepreneurial.orientation ~ c*Jobsecurity + d*Entrepreneurial.self.efficacy + e*hardrecoverytimesemployees + ihsprofit + ihspgrowth + lpu + PersoneelsklasseBTW_O1 + tenure+ wwjobsecurity + Need.for.Achievement

Indirect effect via SJS := a*c

Indirect effect via ESE := b*d

Total effect := a*c + b*d + e

'

fitre<- sem(modelre, data = maindf)

summary(fitre, standardized=TRUE)

modelcpbc <- '

Jobsecurity ~ a*hardcrisistimesprofit

Entrepreneurial.self.efficacy ~ b*hardcrisistimesprofit

Entrepreneurial.orientation ~ c*Jobsecurity + d*Entrepreneurial.self.efficacy + e*hardcrisistimesprofit

Indirect effect via SJS := a*c

Indirect effect via ESE := b*d

Total effect := a*c + b*d + e

'

fitcpbc<- sem(modelcpbc, data = maindf)

summary(fitcpbc, standardized=TRUE)

modelrpbc <- '

Jobsecurity ~ a*hardrecoverytimesprofit

Entrepreneurial.self.efficacy ~ b*hardrecoverytimesprofit

Entrepreneurial.orientation ~ c*Jobsecurity + d*Entrepreneurial.self.efficacy + e*hardrecoverytimesprofit

Indirect effect via SJS := a*c

Indirect effect via ESE := b*d

Total effect := a*c + b*d + e

'

fitrpbc<- sem(modelrpbc, data = maindf)

summary(fitrpbc, standardized=TRUE)

modelcebc <- '

Jobsecurity ~ a*hardcrisistimesemployees

Entrepreneurial.self.efficacy ~ b*hardcrisistimesemployees

Entrepreneurial.orientation ~ c*Jobsecurity + d*Entrepreneurial.self.efficacy + e*hardcrisistimesemployees

Indirect effect via SJS := a*c

Indirect effect via ESE := b*d

Total effect := a*c + b*d + e

'

fitcebc<- sem(modelcebc, data = maindf)

summary(fitcebc, standardized=TRUE)

modelrebc <- '

Jobsecurity ~ a*hardrecoverytimesemployees

Entrepreneurial.self.efficacy ~ b*hardrecoverytimesemployees

Entrepreneurial.orientation ~ c*Jobsecurity + d*Entrepreneurial.self.efficacy + e*hardrecoverytimesemployees

Indirect effect via SJS := a*c

Indirect effect via ESE := b*d

Total effect := a*c + b*d + e

'

fitrebc<- sem(modelrebc, data = maindf)

summary(fitrebc, standardized=TRUE)

fitcprob<- sem(modelcp, data = maindf, estimator = "MLR")

summary(fitcprob, standardized=TRUE)

fitrprob<- sem(modelrp, data = maindf, estimator = "MLR")

summary(fitrprob, standardized=TRUE)

fitcerob<- sem(modelce, data = maindf, estimator = "MLR")

summary(fitcerob, standardized=TRUE)

fitrerob<- sem(modelre, data = maindf, estimator = "MLR")

summary(fitrerob, standardized=TRUE)

lmjs<-lm(Jobsecurity ~ ihsprofit + ihspgrowth + lpu + PersoneelsklasseBTW_O1 + tenure + wwjobsecurity + Need.for.Achievement, data = completemaindf)

lmese<-lm(Entrepreneurial.self.efficacy ~ ihsprofit + ihspgrowth + lpu + PersoneelsklasseBTW_O1 + tenure+ wwjobsecurity + Need.for.Achievement, data = completemaindf)

lmeo<-lm(Entrepreneurial.orientation ~ ihsprofit + ihspgrowth + lpu + PersoneelsklasseBTW_O1 + tenure+ wwjobsecurity + Need.for.Achievement, data = completemaindf)

completemaindf$ressemjs<-residuals(lmjs)

completemaindf$reslmese<-residuals.lm(lmese)

completemaindf$reslmeo<-residuals.lm(lmeo)

plot(reslmjs, reslmeo, xlab = "Job security residualized", ylab = "Entrepreneurial orientation residualized", pch= c(19-completemaindf$hardcrisistimesprofit), col = c(10-2*completemaindf$hardcrisistimesprofit), ylim = c(-30, 70))

legend("topleft", legend = c("Neutral or better", "Somewhat hard", "Hard", "Very hard"), col = c(8,6,4,2), pch = c(18,17,16,15))

plot(reslmjs, reslmeo, xlab = "Job security residualized", ylab = "Entrepreneurial orientation residualized", pch= c(19-completemaindf$hardrecoverytimesprofit), col = c(10-2*completemaindf$hardrecoverytimesprofit), ylim = c(-30, 70))

legend("topleft", legend = c("Neutral or better", "Somewhat hard", "Hard", "Very hard"), col = c(8,6,4,2), pch = c(18,17,16,15))

plot(reslmjs, reslmeo, xlab = "Job security residualized", ylab = "Entrepreneurial orientation residualized", pch= c(19-completemaindf$hardcrisistimesemployees), col = c(10-2*completemaindf$hardcrisistimesemployees), ylim = c(-30, 70))

legend("topleft", legend = c("Neutral or better", "Somewhat hard", "Hard", "Very hard"), col = c(8,6,4,2), pch = c(18,17,16,15))

plot(reslmjs, reslmeo, xlab = "Job security residualized", ylab = "Entrepreneurial orientation residualized", pch= c(19-completemaindf$hardrecoverytimesemployees), col = c(10-2*completemaindf$hardrecoverytimesemployees), ylim = c(-30, 70))

legend("topleft", legend = c("Neutral or better", "Somewhat hard", "Hard", "Very hard"), col = c(8,6,4,2), pch = c(18,17,16,15))

plot(reslmese, reslmeo, xlab = "Entrepreneurial self-efficacy residualized", ylab = "Entrepreneurial orientation residualized", pch= c(19-completemaindf$hardcrisistimesprofit), col = c(10-2*completemaindf$hardcrisistimesprofit), xlim = c(-150, 250))

legend("topright", legend = c("Neutral or better", "Somewhat hard", "Hard", "Very hard"), col = c(8,6,4,2), pch = c(18,17,16,15))

plot(reslmese, reslmeo, xlab = "Entrepreneurial self-efficacy residualized", ylab = "Entrepreneurial orientation residualized", pch= c(19-completemaindf$hardrecoverytimesprofit), col = c(10-2*completemaindf$hardrecoverytimesprofit), xlim = c(-150, 250))

legend("topright", legend = c("Neutral or better", "Somewhat hard", "Hard", "Very hard"), col = c(8,6,4,2), pch = c(18,17,16,15))

plot(reslmese, reslmeo, xlab = "Entrepreneurial self-efficacy residualized", ylab = "Entrepreneurial orientation residualized", pch= c(19-completemaindf$hardcrisistimesemployees), col = c(10-2*completemaindf$hardcrisistimesemployees), xlim = c(-150, 250))

legend("topright", legend = c("Neutral or better", "Somewhat hard", "Hard", "Very hard"), col = c(8,6,4,2), pch = c(18,17,16,15))

plot(reslmese, reslmeo, xlab = "Entrypreneurial self-efficacy residualized", ylab = "Entrepreneurial orientation residualized", pch= c(19-completemaindf$hardrecoverytimesemployees), col = c(10-2*completemaindf$hardrecoverytimesemployees), xlim = c(-150, 250))

legend("topright", legend = c("Neutral or better", "Somewhat hard", "Hard", "Very hard"), col = c(8,6,4,2), pch = c(18,17,16,15))
