# Supplementary material for: When the going gets tough, the entrepreneurs get less entrepreneurial?
Source: PLoS One. 2023 Dec 22;18(12):e0290253. doi: 10.1371/journal.pone.0290253 (PMC10745155; doi:10.1371/journal.pone.0290253)
Supplement: Attachment — Submitted filename: Response to Reviewers.docx [file pone.0290253.s002.docx]

**Response to reviewers**

PONE-D-23-02387

When the going gets tough, the entrepreneurs get less entrepreneurial?

*Thank you for the opportunity to revise and resubmit our paper. Please find our responses to your suggestions indented and in italics.*

Editor’s comments:

Please expand the originality and significance of contribution in introduction section by adding more arguments about the contribution to existing knowledge.

*We added an explicit mention of the rich data we show an analysis of.*

IN the methods section there are some technical details that could improve readability of the paper.

*We have clarified the technical details where the reviewers noticed unclarity.*

In conclusions you need to expand implications of your study and also add limitations which basically are missing.

*We have clarified the implications in the conclusions by moving the more nuanced and involved discussion to the new limitations subsection in the discussion section. Now the conclusion really drives home the main message.*

*Furthermore, the discussion section is now reorganized into an implications subsection, where expanded implications are mentioned.*

5. Review Comments to the Author

Reviewer #1: METHOD
Table 1. Variables and measures show that rich data sources are used. This can be considered a strength of the research.
*Thank you for appreciating the valuable and unique richness of our data. This comment inspired	us to sell this richness explicitly in the introduction.*

In line 274, we see that 5 companies in the sample have more than 50 employees. In the sample, you said that most of them are less than 5 employees. How many, please specify.
Sample. As part of an ongoing consulting project involving entrepreneurs and their SMEs, we asked 532 entrepreneurs in great detail about their venture, as an enterprise, and about themselves as an individual, a couple of weeks later. In the venture questionnaire, we measured EO and satisfaction with performance. In the individual survey, we measured our pair of mediators: ESE and SJS. Thus, measures of concepts directly linked in our conceptual model are never sourced from the same survey. This time lag substantially reduces the threat of common-method variance, probably to (close to) zero [49]. The sample contains entrepreneurs and their ventures from Flanders (the Dutch-speaking part of Belgium), from 55 three-digit industries. All but five ventures have less than 50 employees, and many even have less than five employees.

In line 388, we see that 1 company in the sample has more than 50 employees.
Sixth, larger ventures should be more buffered against hard times, so post-crisis recovery is less likely to be very dissatisfactory, and (sense of) job security should be greater. Very large ventures are often more inert, and thus less entrepreneurially oriented. However, all but one ventures in our sample have fewer than 50 employees. With that range, it may be that this covariate is not impactful.

*We have corrected this inconsistency (also in line 578). There are 5 companies with between 50 and 100 employees, and one company with 100-200 employees.*

*We now also specify the number of companies with less than 5 employees (104).*

Structural equation models estimated with the lavaan 396 packages in R were used in the research. Therefore, giving the script in Appendix H is essential for the reproducibility of the study.
*Thank you for appreciating our efforts to make the study as reproducible as we can.*

RESULTS
Giving scatter diagrams as an attachment is healthier for reading the text. Otherwise, there are too many shapes. This can make the text difficult to read.

*We agree with this concern but propose a different solution. Our plan is to ensure readability at the copyedit stage by indicating that the scatter diagrams should be published at half the size of regular figures. But we are open to other suggestions.*

DISCUSSION

1-Main results are compatible with the crisis imprinting theory,
2- The behavioral theory of the firm is included in H5alt.
3- Another theory that may be related is the 'trial by fire' model
4- The second aspect is a causal mechanism, which is more developed under the banner of "the red queen effect."
Including the first two theories in the introduction and literature and the third and fourth theories in the discussion creates confusion. However, when the discussion section is thoroughly read, it is seen that the justification is well done.

*Thank you for appreciating the justification, and thank you for reading thoroughly. We reorganized the discussion to reduce confusion.*

CONCLUSION
Please give abbreviations where they are first used: for example: "sense of job security (SJS)" on line 603. Please check all abbreviations in the article.
The indirect effects of hard times on EO via ESE and SJS are weak. The reasons for that weakness differs between them. The indirect effect via ESE is weak due to the weak effect of some types of hard times on ESE. Hard times during crisis have a weaker effect on ESE than hard times after the crisis, which suggests that our persistence premise may be weak for ESE. The indirect effect via sense of job security (SJS) is weak because, although hard times do reduce SJS, SJS is only weakly related to EO. A possible explanation for this is that the evidence from employee samples that SJS influences EO only partially applies to the entrepreneur context.

*Thank you for pointing this out. We have corrected this and also doublechecked all other abbreviations.*

Please proofreading the entire article.

*We carefully doublechecked the article for any spelling or grammar issues.*

Reviewer #2: Thank you for providing to me the opportunity to review the article. I think that there are few studies that examine entrepreneurship during the crisis, thereby, this article makes contribution to the literature. There is no limitations and future suggestions section, I recommend to authors to write as sub-section within the discussion section.

*Thank you for recognizing our contribution to the literature. We have added a limitations and future research subsection as recommended. This comment pushed us to reorganize our discussion and conclusion into a more focused format, improving the paper.*

2. In your Data Availability statement, you have not specified where the minimal data set underlying the results described in your manuscript can be found. PLOS defines a study's minimal data set as the underlying data used to reach the conclusions drawn in the manuscript and any additional data required to replicate the reported study findings in their entirety. All PLOS journals require that the minimal data set be made fully available. For more information about our data policy, please see <http://journals.plos.org/plosone/s/data-availability>.

Upon re-submitting your revised manuscript, please upload your study’s minimal underlying data set as either Supporting Information files or to a stable, public repository and include the relevant URLs, DOIs, or accession numbers within your revised cover letter. For a list of acceptable repositories, please see <http://journals.plos.org/plosone/s/data-availability#loc-recommended-repositories>. Any potentially identifying patient information must be fully anonymized.

Important: If there are ethical or legal restrictions to sharing your data publicly, please explain these restrictions in detail. Please see our guidelines for more information on what we consider unacceptable restrictions to publicly sharing data: <http://journals.plos.org/plosone/s/data-availability#loc-unacceptable-data-access-restrictions>. Note that it is not acceptable for the authors to be the sole named individuals responsible for ensuring data access.

*We have paid extra attention to being as specific as possible about our data availability.*
